# Pitavastatin sensitizes the EGFR-TKI associated resistance in lung cancer by inhibiting YAP/AKT/BAD-BCL-2 pathway

**DOI:** 10.1186/s12935-024-03416-z

**Published:** 2024-06-28

**Authors:** Jie Liu, Jialei Fu, Ping Fu, Menghan Liu, Zining Liu, Bao Song

**Affiliations:** 1 Cancer Center, Shandong Public Health Clinical Center, Shandong University, Jinan, China; 2 Shandong Academy of Chinese Medicine, Jinan, China; 3 Department of Chemotherapy, Jinan Zhangqiu District People’s Hospital, Jinan, China; 4 Clinical Medical College, Shandong First Medical University, Jinan, China; 5 Department of Nuclear Medicine, Shandong First Medical University and Shandong Academy of Medical Sciences, The Third Affiliated Hospital of Shandong First Medical University, Jinan, China; 6 Shandong Provincial Key Laboratory of Radiation Oncology, Shandong Cancer Hospital and Institute, Shandong First Medical University and Shandong Academy of Medical Sciences, Jinan, China

**Keywords:** Pitavastatin, EGFR-TKI, Drug resistance, Lung cancer, YAP

## Abstract

**Background:**

Despite effective strategies, resistance in EGFR mutated lung cancer remains a challenge. Metabolic reprogramming is one of the main mechanisms of tumor drug resistance. A class of drugs known as “statins” inhibit lipid cholesterol metabolism and are widely used in patients with cardiovascular diseases. Previous studies have also documented its ability to improve the therapeutic impact in lung cancer patients who receive EGFR-TKI therapy. Therefore, the effect of statins on targeted drug resistance to lung cancer remains to be investigated.

**Methods:**

Prolonged exposure to gefitinib resulted in the emergence of a resistant lung cancer cell line (PC9GR) from the parental sensitive cell line (PC9), which exhibited a traditional EGFR mutation. The CCK-8 assay was employed to assess the impact of various concentrations of pitavastatin on cellular proliferation. RNA sequencing was conducted to detect differentially expressed genes and their correlated pathways. For the detection of protein expression, Western blot was performed. The antitumor activity of pitavastatin was evaluated in vivo via a xenograft mouse model.

**Results:**

PC9 gefitinib resistant strains were induced by low-dose maintenance*.* Cell culture and animal-related studies validated that the application of pitavastatin inhibited the proliferation of lung cancer cells, promoted cell apoptosis, and restrained the acquired resistance to EGFR-TKIs. KEGG pathway analysis showed that the hippo/YAP signaling pathway was activated in PC9GR cells relative to PC9 cells, and the YAP expression was inhibited by pitavastatin administration. With YAP RNA interference, pAKT, pBAD and BCL-2 expression was decreased, while BAX expression as increased. Accordingly, YAP down-regulated significantly increased apoptosis and decreased the survival rate of gefitinib-resistant lung cancer cells. After pAKT was increased by SC79, apoptosis of YAP down-regulated cells induced by gefitinib was decreased, and the cell survival rate was increased. Mechanistically, these effects of pitavastatin are associated with the YAP pathway, thereby inhibiting the downstream AKT/BAD-BCL-2 signaling pathway.

**Conclusion:**

Our study provides a molecular basis for the clinical application of the lipid-lowering drug pitavastatin enhances the susceptibility of lung cancer to EGFR-TKI drugs and alleviates drug resistance.

**Supplementary Information:**

The online version contains supplementary material available at 10.1186/s12935-024-03416-z.

## Introduction

Gene mutation in human epidermal growth factor receptor (EGFR) has a substantial impact on non-small cell lung cancer (NSCLC). Approximately 50% of Asian NSCLC patients and 15% of Caucasian population are affected with activated mutations in the EGFR gene [1], comprising deletions, point mutations, and insertions, in exons 18–25 of the EGFR gene [2]. EGFR tyrosine kinase inhibitors (TKIs) -targeted therapy holds a strong inhibitory action on lung cancer, with fewer adverse effects compared with traditional chemotherapeutic drugs. However, the monotherapy of EGFR-TKIs still faces great challenges. Medical evidence suggests that 30% of patients with EGFR mutations develop secondary drug resistance after 12–18 months of treatment with an EGFR-TKI monotherapy [3]. Acquired drug resistance mechanisms were mainly EGFR T790M mutations or T797S mutations, in addition, activation of downstream signaling (i.e. amplified MET, mutations in PIK3CA, and activation of AXL) non-genetic resistance mechanisms are also involved [4, 5]. The third generation EGFR TKI osimertinib can treat the acquired EGFR T790M mutation; however, it does not affect other types of acquired resistance mutations. Therefore, finding an alternative treatment strategy for the acquisition of TKI resistance in lung cancer is critical.

During tumor development, tumor cells reprogram their energy metabolism pathways to resist the external world. Metabolic reprogramming is closely related to tumor drug resistance. Various signaling mechanisms associated with key regulators of lipid metabolism can influence cell commitment, the expression pattern of a gene, stress-mediated pathways, or the microenvironment of the tumor, thereby inducing tumor growth [6]. Studies have shown that lipid synthesis is further increased in tumor-resistant cells. Lipid metabolism can produce a large number of intermediates, such as mevalonate (MVA), isoprenoids, etc., and its altered activity may affect its downstream signaling pathway disorder [7]. For example, farnesyl pyrophosphate (FPP) and geranyl pyrophosphate (GGPP) are essential for the prenylation of small GTPases post-translationally, and have essential roles in a number of intracellular signaling cascades [8, 9]. In particular, GGPP activates Yes-associated protein (YAP) by preventing its phosphorylation and supporting its accumulation inside the nucleus. YAP serves as a chief downstream effector molecule for the Hippo pathway, and acts as an important regulator of transcription by the over-expression of pro-growth and downregulation of pro-apoptotic genes. YAP has been found to be a key oncogene in various types of cancer [10–13]. In particular, YAP is closely associated with resistance to conventional chemotherapeutic drugs, and it is also closely related to targeted therapies such as BRAF/MEK/ EGFR [14–17].

Statins are cholesterol-lowering drugs, but in recent years, their effects have become increasingly apparent, including anti-inflammatory, immunomodulation, neuroprotection, improved bone metabolism, and antitumor effects [18–20]. Growing rate of preclinical and epidemiological evidence describes an inverse relation between statins, potent MVA biosynthesis pathways inhibitors, and mortality resulting from certain cancers (colon, prostate, liver, breast, hematological malignancies and lung cancer) [21–27]. A study documented that statins present improved therapeutic effects and enhanced survival of lung cancer patients receiving EGFR-TKI treatment [28]. Therefore, the study aims to investigate whether pitavastatin can suppress YAP signaling in EGFR mutant lung cancer, anti-resistance to acquired EGFR TKIs resistance and its mechanism. In the present study, gefitinib-resistant NSCLC cell lines were developed and we found that pitavastatin could alleviate drug resistance in the geftinib-resistant cells and the effect is achieved through the YAP/AKT/BCL-2 pathway.

## Materials and methods

### Cell lines, primary cells and drugs

Human NSCLC cell lines (A549, H1299 and PC9) and were procured from the Chinese Academy of Sciences (Shanghai, China) and cultured in RPMI-1640 Medium enriched with 10% FBS (Gibco, USA). The lung fibroblast cell MRC-5 was procured and cultured in in special medium (Haixing Biosciences, Suzhou,China). Increased concentrations of gefitinib (ranging from 0.1 to 10 μM) were exposed to the parental gefitinib-sensitive PC9 cells over 6 months to establish a PC9GR cell line (PC9 gefitinib resistant cells). Pitavastatin and SC79 were purchased from GLPBio (ontclair, California). Gefitinib was obtained from Beyotime (Shanghai, China).

### Cell proliferation assay

In 96-well microtiter plate, 2000 cells/well were seeded in 100 μL culture medium. Pitavastatin and/or gefitinib were added to each well as indicated and incubation was proceeded for 24–72 h. In each well, 10 μL volume of CCK-8 reagent (5 mg/mL) was added. The OD (absorbance) was noted by ThermoFisher Spectrophotometer 1510 (Molecular Devices, Inc.) at 450 nm. The antiproliferative activity related to gefitinib and pitavastatin was analyzed by determining the combination index (CI), which was analyzed via CompuSyn software (ComboSyn, USA). CI < 1 and CI > 1 indicate a synergistic and antagonistic effect [29].

### Cell apoptosis

For cell apoptosis analysis, approximately 3 × 10^5^ cells/well were grown in 6-well plates and treated with the defined gefitinib and/or pitavastatin concentrations. Then, cells were trypsinized after 48–72 h and labeled with FITC-Annexin V (BD biosciences, USA) in the dark for 15 min. The stained population of cells was analyzed by the FACS Calibur instrument (Becton Dickinson, USA).

### Nude mouse xenograft

The female BALB/c-nu mice, which were 4–6 weeks old (obtained from Beijing HFK Bioscience, China), were administered 5 × 10^6^ PC9 and PC9GR cells resuspended in 100 μL PBS via subcutaneous injection. When the cells attained an approximate size of − 100 mm^3^, the mice with cells were split randomly into four groups: Control (DMSO), gefitinib (2 mg/kg for PC9 and PC9GR), pitavastatin (4 mg/kg), gefitinib and pitavastatin. The treatment lasted 3–8 weeks and was administered three times per week via intraperitoneal injection. The volume of the tumor was determined via the following formula: tumor volume (mm^3^) = 1/2 × (length × square width) and tumor size was assessed two times per week. Following the end of the investigation, the tumors were extracted from the sacrificed rats and weighed. All animal-related protocols were approved based on the Animal Management Committee of Shandong Cancer Hospital and Institute.

### Targeted genetic sequencing (TGS) and RNA sequencing

Targeted genetic sequencing was performed using CancerSCAN panel (Covaris,MA) including the 10 genes linked to lung cancer on PC9 and PC9GR cells. A whole transcriptome assay was carried out via RNA sequencing on 4 distinct cell groups: parental PC9 cells (Group A), PC9 cells treated with 5 μM pitavastatin for 48 h (Group B), PC9GR cells (Group C), and PC9GR cells treated with 5 μM pitavastatin for 48 h (Group D). For each group, three separate samples were grown and treated independently. Extracted whole RNA was submitted for sequencing to Biomarker Technology Co., Ltd. (Beijing, China). Subsequently, enrichment analyses of pathways and gene ontologies (GO) were carried out on the DEGs. A pathway map from KEGG (https://www.genome.jp/kegg/) was downloaded.

### Western blotting

Western blot analysis was conducted by using whole cell lysates prepared in RIPA buffer (1 × PBS, 0.1% sodium dodecyl sulfate, 0.5% sodium deoxycholate, 1% NP-40). The preceding buffer was supplemented with 1 mM PMSF, 10 mM β-glycerophosphate, 10 mM NaF, and a 1 × protease inhibitor cocktail (Roche, Indianapolis, IN). Before transferring onto nitrocellulose membranes, equivalent quantities of total proteins were determined by 10–12% SDS-PAGE. Membranes were blocked with 5% dried skim milk and kept with primary antibody for 1 h at 25 ℃. The antibodies utilized included rabbit antibodies targeting human EGFR, pEGFR, YAP, AKT, pAKT, BAD, pBAD, BCL-2, BAX and β-actin (all 1:1000; Beytime Biotechnology, China). Using Super Signal West Pico Chemiluminescent Substrate (Pierce Biotechnology), the signals were observed subsequent to the rinsing process. For each sample, a minimum of three Western blots were executed.

### Real-time RT-PCR

TRIzol reagent (Invitrogen) was utilized to extract total RNAs from the cultured cells, which were subsequently reverse-transcribed into cDNA via the PrimeScript reverse transcriptase reagent kit. To perform real-time RT-PCR, cDNA was amplified on the QuantStudio 6Flex system (appliedbiosystems, MD, USA) using a SYBR Premix Ex Taq reagent (TaKaRa; DRR041). Each reaction was carried out in triplicate. A list of the primers used during real-time PCR was provided in the Supplementary Materials.

### DNA plasmids, siRNA, and its sequences and transfection

The control, human plasmids pcDNA3.1-YAP and YAP-specific siRNAs were all provided by GenePharma (Shanghai, China). The target sequences comprised the following: CUGCCACCAAGCUAGAUAATT siYAP1; GCCAGUACUGAUGCAGGUATT siYAP2. Plasmids were transfected into cells in line with the manufacturer’s recommendations via Lipofectamine 2000 (Invitrogen, USA). The cells transfected with 5 nmol/L of siRNA, and scrambled negative control using Lipofectamine 2000 were incubated for 48–72 h.

### Immunohistochemical (IHC) staining

For immunohistochemistry, paraffin-embedded sections were prepared from the fixed xenograft tumor tissues. Antigen in specimens was retrieved subsequent to their routine dewaxing and hydration. Tissue sections were blocked to prevent the activity of endogenous peroxidase, kept with goat serum, and subsequently labeled with anti-Ki67 and BCL-2 antibodies (Servicebio Technology, China). HRP-conjugated anti-rabbit IgG and DAB peroxidase substrate were utilized for secondary staining. The photographs were captured via phase contrast microscope (Leica, Germany).

### Statistical analysis

To conduct statistical analyses, the SPSS20.0 software (IBM, USA) was employed. Different statistical tests including the T-test, the chi-square test, and the Spearman rank correlation were utilized to analyze counting data and compare measurement data. The data are reported in mean ± SD. In all experimental examinations, the level of significance was set at *p* < 0.05.

## Results

### Comparison of EGFR TKI-resistant and sensitive lung *cancer* cells

**Fig. 1:**
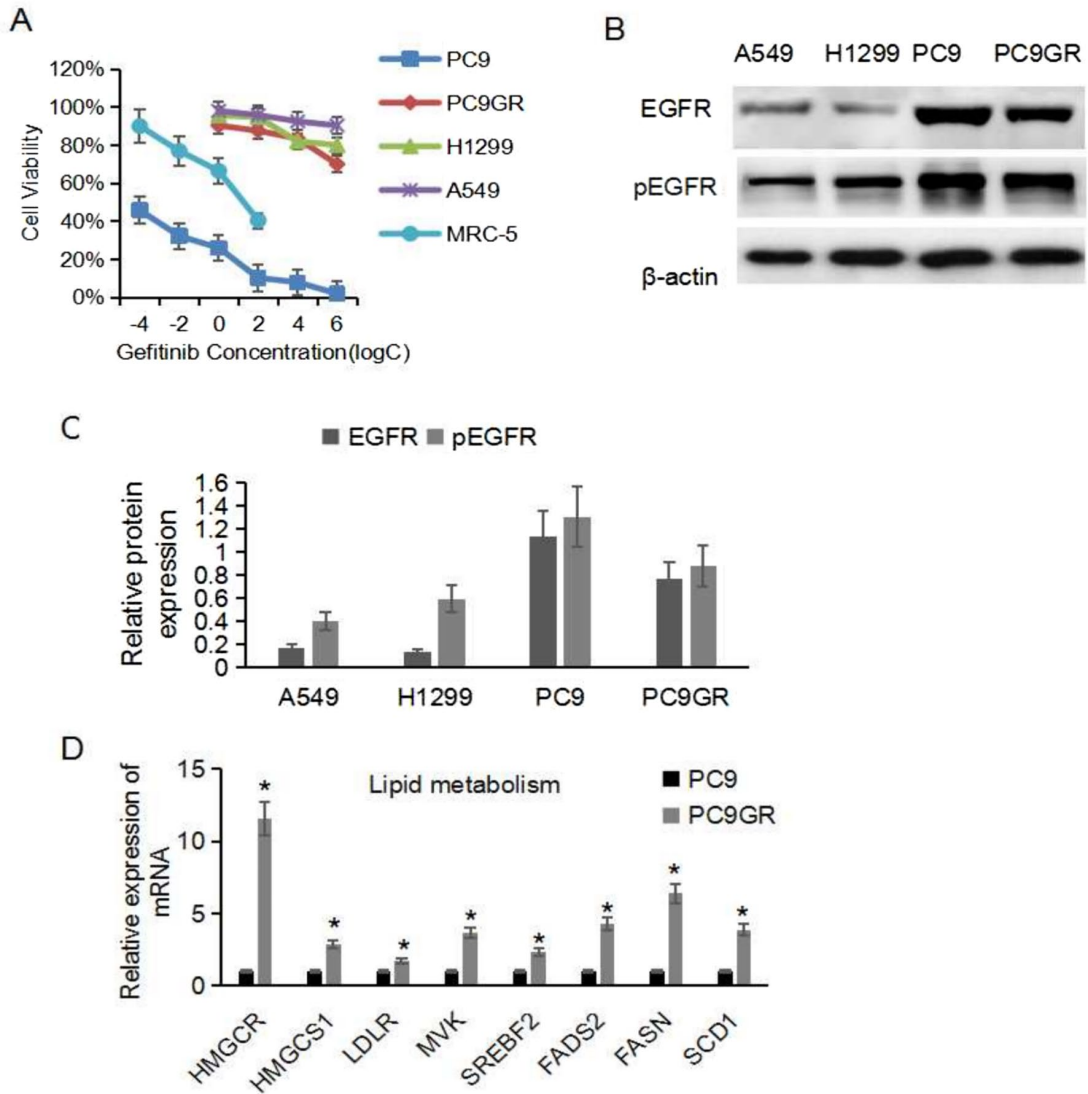
A Comparative analysis of sensitive and EGFR TKI-resistant lung cancer cells.** A** Sensitivity of A549, H1299, PC9 and PC9GR cells to gefitinib. **B** Western blot of EGFR and pEGFR expression in A549, H1299, PC9 and PC9GR cells. **C** Relative protein levels of pEGFR in A549, H1299, PC9 and PC9GR cells. **D** The expression of lipid metabolism related genes was analyzed by qRT-PCR in PC9 and PC9GR cells(^*^P < 0.05)

According to the Cancer Cell Line Encyclopedia (CCLE) database, lung cancer cell lines have different genetic backgrounds. A549 and H1299 cells harbor wild-type EGFR; PC9 cells harbor EGFR exon 19 deletion. After 6 months of geftinib treatment, geftinib-resistant NSCLC cell line PC9GR was established from the geftinib-sensitive PC9 cell line. We confirmed that both PC9 and PC9GR cells possessed a 15-base deletion in exon 19 of EGFR via TGS. Notably, the PC9GR cells acquired an NRAS Gln611Lys mutation that was not found in their parental PC9 cells. These lung adenocarcinoma cells were treated with gefitinib (0 ~ 40 μmol/L) for 48 h. The IC50 values of the lung cancer cells for gefitinib were quantified and presented in Fig. 1A, which were 21.3, 9.5, and 0.17 μmol/L for A549, H1299, PC9, respectively. The sensitivity of PC9 cells to gefitinib was far greater than that of other cell lines. The IC50 value for geftinib in PC9GR cells was 10.8 μmol/L, which was 63-fold higher than that in PC9 cells. For MRC-5 lung fibroblasts, IC50 of gefitinib was 3.6 μmol/L, indicating that gefitinib had certain damage to normal lung tissue. PC9GR cells exhibited a slight decrease in expression of pEGFR compared to PC9 cells (Fig. 1B–C). The detection of lipid metabolism-related genes in PC9GR cells and parental cells showed that cholesterol and fatty acid metabolism genes HMGCR, LDLR, MVK, SREBP2, FADS2, FASN and SCD1 were increased to a significant degree (Fig. 1D).

### Pitavastatin inhibited proliferation, promoted apoptosis and restrained acquired gefitinib resistance

**Fig. 2:**
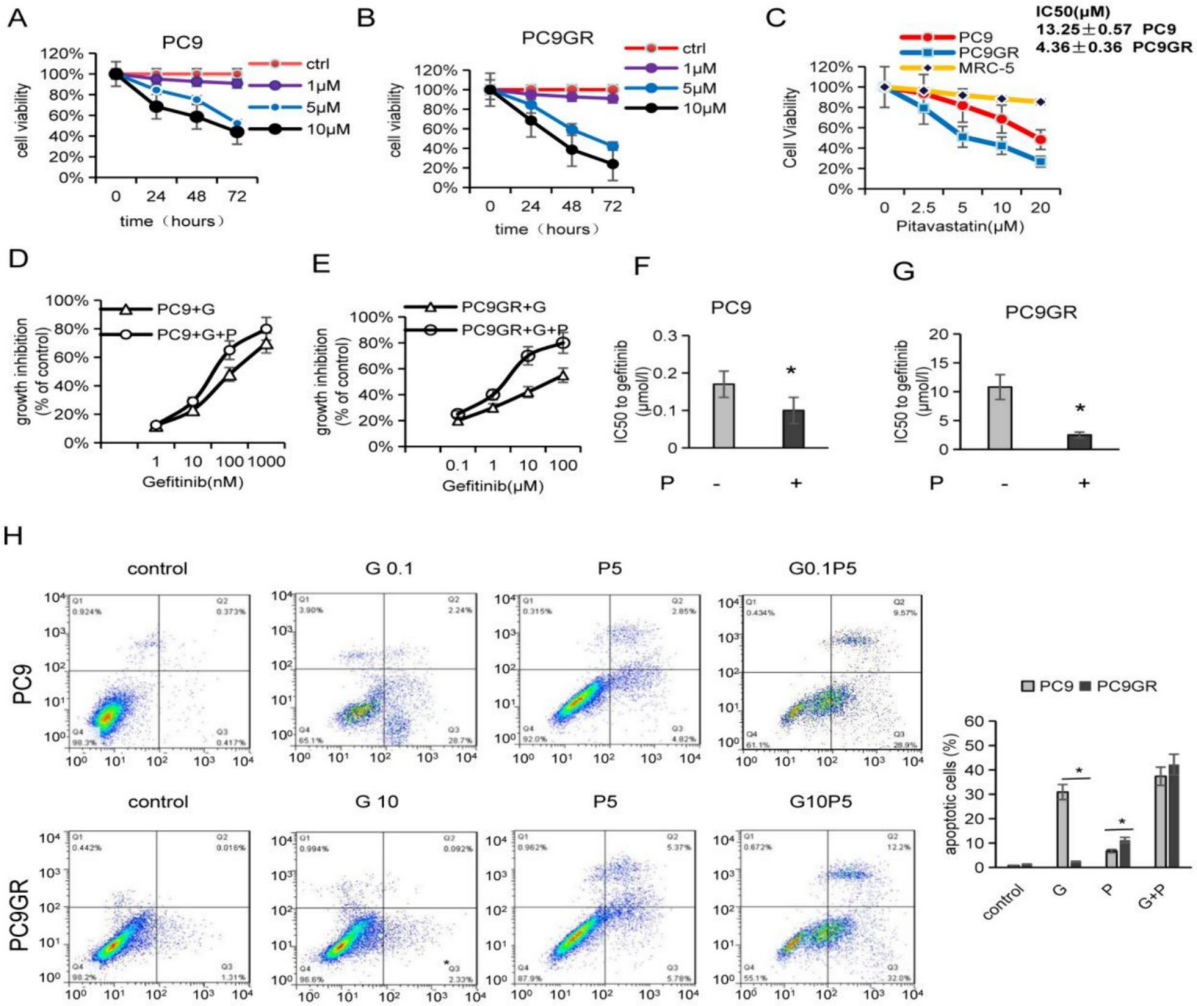
Pitavastatin accelerated apoptosis and suppressed propagation of lung cancer cells.** A**–**B** PC9 and PC9GR cells were treated with defined pitavastatin concentrations, cell viability was measured by CCK-8 assay. **C** Comparison of IC50 values of pitavastatin for PC9 and PC9GR cells. **D**–**E** Effect of combined treatment on growth inhibition, the dose of pitavastatin was 10 μM in PC9 and 5 μM in PC9GR. Cells were treated with the drugs for 48 h, and then cell growth was assessed by CCK-8 assay. **F-G** IC50 values to gefitinib of PC9 and PC9GR cells, with or without pitavastatin treatment. **H** Pitavastatin combined with gefitinib significantly enhanced the apoptosis of PC9 and PC9GR cells. *p < 0.05

We attempted to observe if pitavastatin could enhance the growth inhibition effect of gefitinib on lung cancer cells. CCK-8 assay showed that pitavastatin inhibited cell viability in a dose-time dependent manner in PC9 gefitinib-sensitive cells and PC9 gefitinib-resistant cells (Fig. 2A–B). The drug-resistant cells were more sensitive to pitavastatin and the IC50 value decreased significantly, from13.25 ± 0.57 μM (PC9 cells) to 4.36 ± 0.36 μM (Fig. 2C). The synergistic impact of gefitinib and pitavastatin on PC9 and PC9GR were evaluated. The cells were simultaneously treated with gefitinib (10–1000 nmol/l for PC9 and 1–100 µmol/l for PC9GR cells) and pitavastatin (0.1–100 µmol/l for both cell lines) for 48 h. In EGFR-TKI-sensitive PC9 cells, the synergistic effect was very weak (Fig. 2D), with a mean CI of 0.9. However, in PC9GR cells, there was a synergistic effect between the two drugs (Fig. 2E), with a mean CI of 0.59. It also showed that the IC50 of gefitinib in PC9GR cells decreased more significantly than in PC9 cells treated with pitavastatin (Fig. 2F–G). Next, the effect of pitavastatin on apoptosis of lung cancer cells was tested. As revealed in Fig. 2H, the rates of apoptosis in PC9 and PC9GR cells induced by gefitinib alone (0.1 μM for PC9 and 10 μM for PC9GR) were 30.9 and 2.2%, and by pitavastatin 5 μM alone were 7.6% and 11.1%, respectively. After combining pitavastatin and gefitinib, apoptosis in PC9 and PC9GR cells was 38.4% and 44.2%, respectively. These findings suggested that concurrent exposure to pitavastatin and gefitinib resulted in a synergistic effect in gefitinib-resistant cells and a weak synergistic effect in gefitinib-sensitive NSCLC cells.

### Pitavastatin overcame and delayed acquired EGFR TKIs resistance in lung *cancer* in vivo

**Fig. 3:**
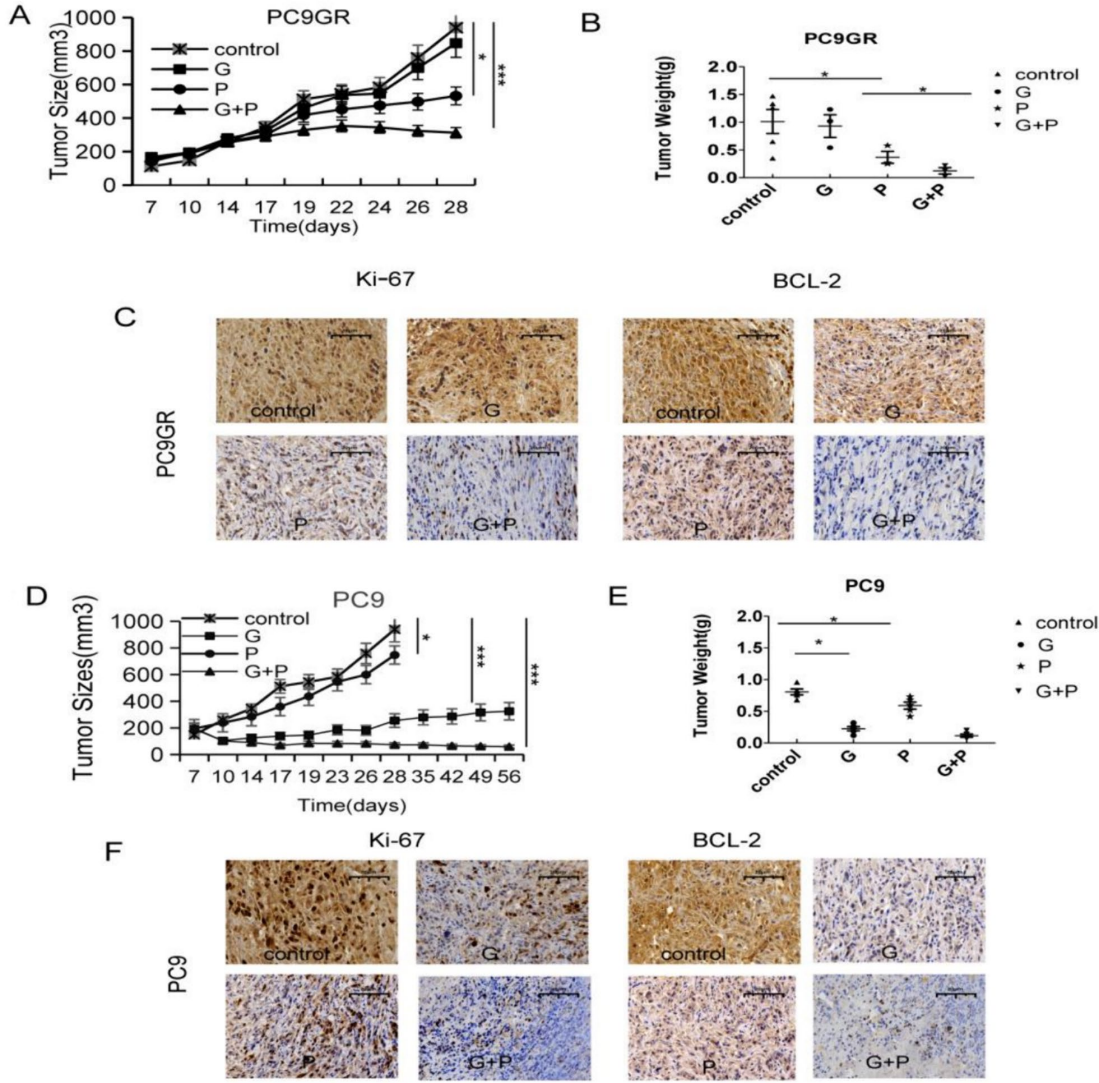
Pitavastatin overcame and delayed gefitinib resistance in lung cancer cells from xenograft mice. The mice were subcutaneously injected with PC9 and PC9GR cells, divided into 4 groups and given the following treatments: DMSO, pivastatin, gefitinib, pivastatin, and gefitinib. **A** and **D** The size of the tumors in nude mice were assessed at 2–4 days. Mean ± SD values (mm^3^) values for each group's tumor volume were calculated. **B** and** E** Tumor weight in diferent groups were shown. **C** and** F** Immunohistochemistry-determined protein levels of Ki-67 and BCL-2 in the tumor tissues of mice in the different treated groups. n = 5 in each group.*p < 0.05, ** p < 0.01, *** p < 0.001

To explore the antitumor activity of pitavastatin in vivo, nude mouse PC9 and PC9GR xenografts were constructed. As expected, there was some significant reduction in tumor volume and weight in the pitavastatin group in the PC9GR transplant tumor model, and no significant change in tumor volume after gefitinib alone. Compared with gefitinib alone, the combination of pitavastatin and gefitinib substantially suppressed tumor growth in PC9GR cells (Fig. 3A–B). Immunohistochemical results showed that G + P substantially suppressed tumor growth and enhanced its apoptosis by decreasing Ki-67 and BCL-2 levels (Fig. 3C). In PC9 transplant tumor models, pitavastatin slightly suppressed tumor growth, while gefitinib alone or G + P resulted in significant regression of xenografted tumors (Fig. 3D–E). As shown in Fig. 3D, gefitinib failed to stop PC9 tumor recurrence after 4–5 weeks of treatment, indicating the emergence of new resistance, whereas G + P effectively inhibited tumor growth for approximately 8 weeks. In addition, Immunohistochemical test results showed that the expression of cell proliferation signal Ki67 and apoptosis index BCL-2 in PC9 were decreased after combined treatment (Fig. 3F). These results indicate that pitavastatin overcomes and delays acquired EGFR TKIs resistance in lung cancer patients.

### Impacts of pitavastatin on the signaling pathway of lung *cancer* cells

**Fig. 4:**
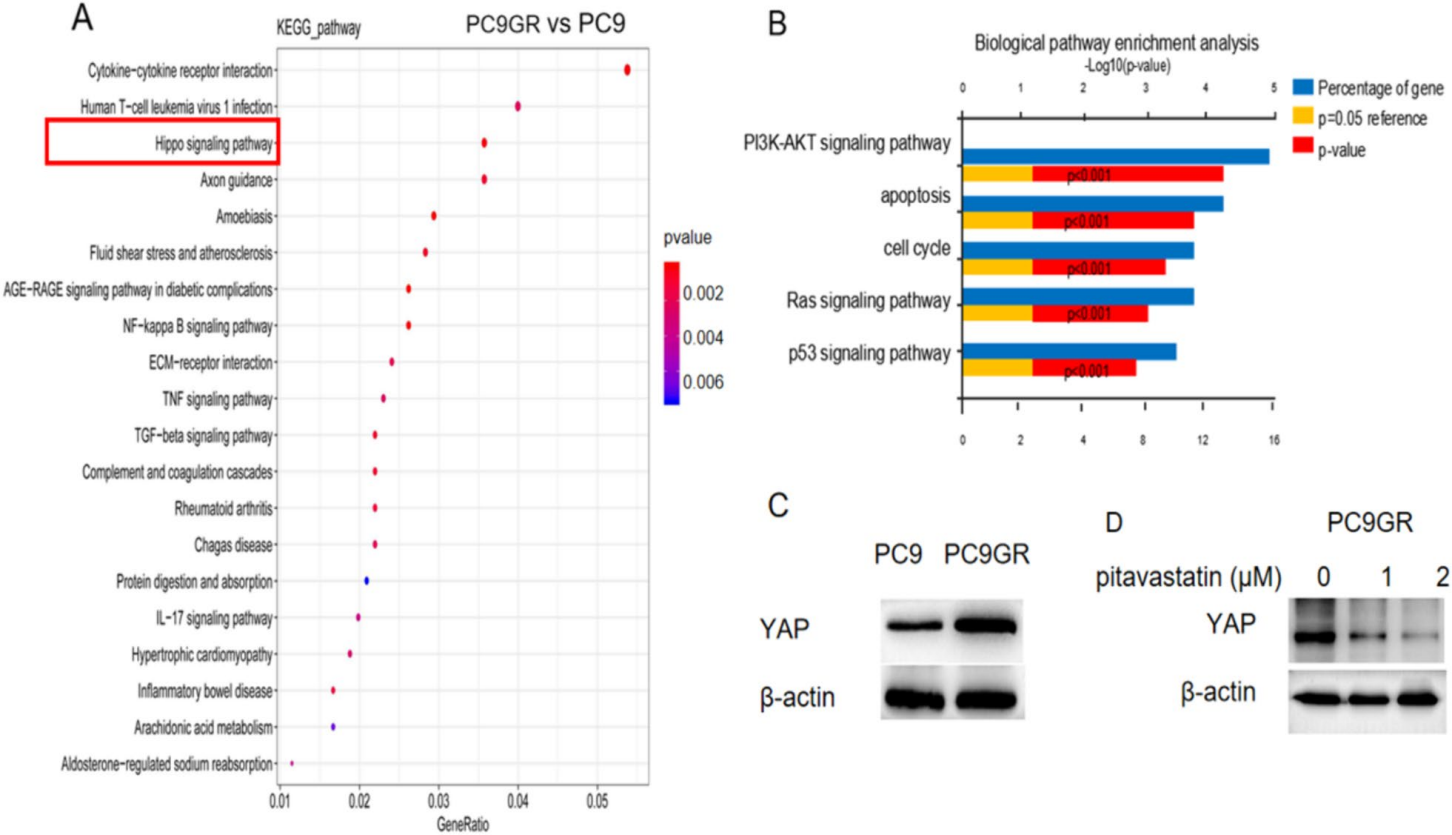
Enrichment of PC9GR cell pathways subsequent to pitavastatin treatment. **A** Enriched KEGG pathway analysis(PC9GR vs. PC9 cells). **B** Enriched biological pathway analysis (Pitavastatin treated PC9GR cells vs. PC9 cells). **C** The protein expression of YAP in PC9 and PC9GR cells. **D** The protein expression of YAP in PC9GR cells with pitavastatin treatment

To understand the mechanism of pitavastatin on lung cancer cells, we used RNA sequencing to detect gene expression differences in PC9 and PC9GR cells. We obtained transcriptomic data of 4 groups of cells: parental PC9 cells (group A), PC9 cells after pitavastatin treatment (group B), parental PC9GR cells (group C), and PC9GR (group D) cells after pitavastatin treatment. The changed signaling pathways are mainly cytokine interaction gene pathway, Hippo signaling pathway, NF-kappa B signaling pathway, AGE-RAGE diabetes complication pathway, etc. These pathways were strongly associated with gefitinib resistance in resistant PC9GR cells (group C) compared to non-resistant PC9 cells (group A) (Fig. 4A). In addition, the transcriptional expression profile also showed that the effect of pitavastatin was mainly concentrated in the PI3K-AKT pathway, apoptosis, cell cycle, RAS pathway, p53 pathway, etc., through the comparison of group C: group D (Fig. 4B). Hippo/YAP signaling pathway was a highly conserved signaling pathway that controls cell growth and organ size by manipulating cell proliferation, division and death. The increased expression of YAP indicates that YAP plays an important role in drug-resistant PC9GR cells (Fig. 4C). Western confirmed that the application of pitavastatin could down-regulate the expression of YAP in PC9 gefitinib resistant lung cancer cells (Fig. 4D).

### Pitavastatin inhibits the proliferation and drug resistance of PC9GR cells by inhibiting YAP signaling pathway

**Fig. 5:**
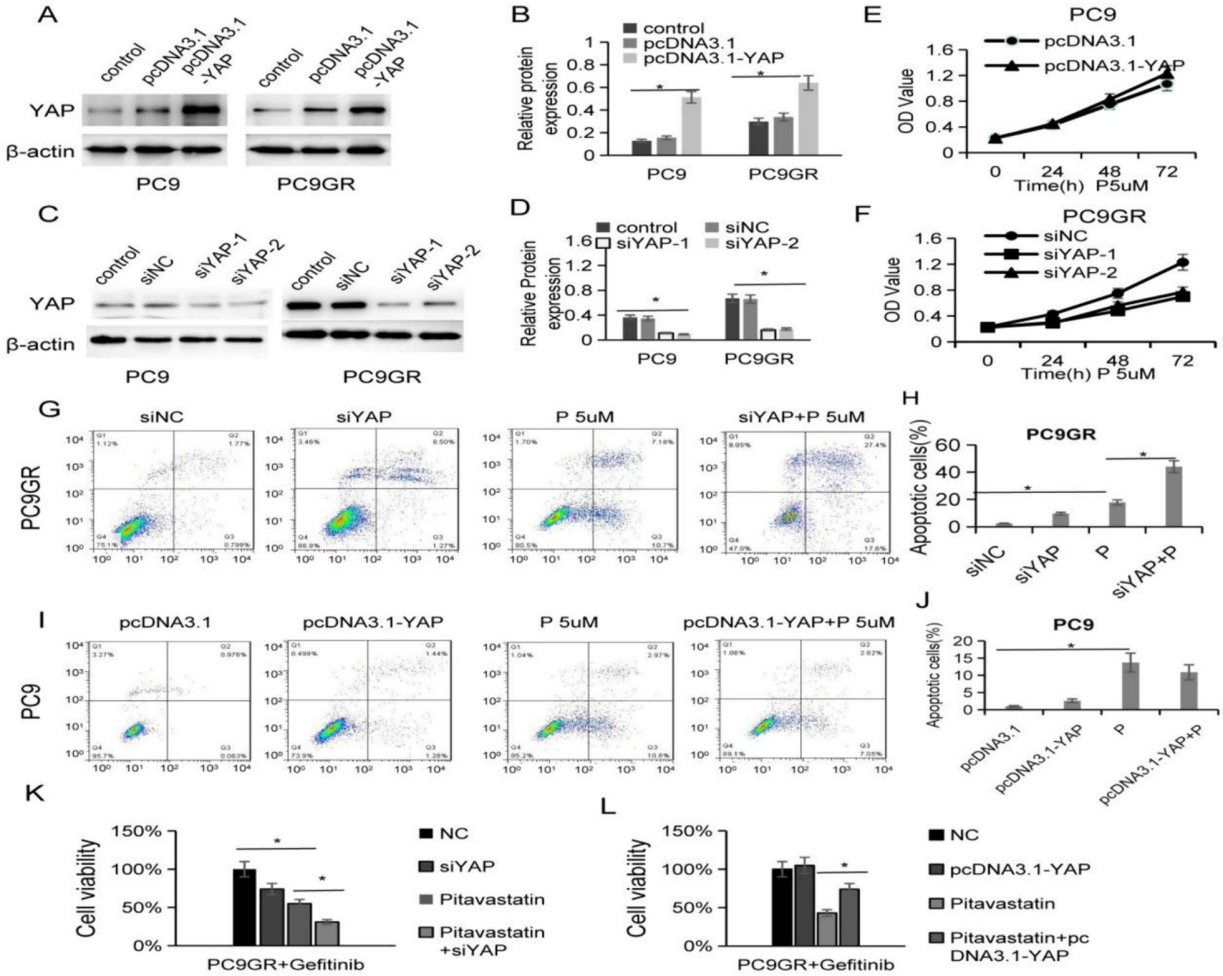
Pitavastatin inhibits proliferation and drug resistance of PC9GR cells by regulating YAP pathway.** A**–**B** YAP protein expression increased in PC9 and PC9GR cells with YAP overexpression. **C-D** YAP protein expression decreased in YAP siRNA PC9 and PC9GR cells. **E** Cell proliferation of PC9 cells with pcDNA3.1-YAP after pitavastatin treatment. **F** Cell proliferation of PC9GR cells with siYAP after pitavastatin treatment. **G-J** Cell apoptosis of PC9GR cells with siYAP and PC9 cells with pcDNA3.1-YAP and pitavastatin treatment. **K**–**L** Effect of siYAP or pcDNA3.1-YAP combined with pitavastatin on cell proliferation after PC9GR gefitinib treatment. (*p < 0.05)

To examine the possible involvement of pitavastatin in the control of the YAP pathway, pcDNA3.1-YAP plasmid and YAP small interfering RNA sequences were transferred into lung cancer cells, and the efficiency of YAP overexpression and knockdown was detected by western blot (Fig. 5A–D). Then the cells were treated with 5μM pitavastatin after YAP overexpression and depletion. It showed that YAP overexpression mildly reduced the inhibition of pitavastatin on PC9 cells, while YAP interference significantly enhanced the inhibition of pitavastatin on PC9GR cell proliferation (Fig. 5E–F). Flow cytometry displayed that the low expression of YAP (siYAP-1 was applied in the follow-up experiment) further significantly enhanced the apoptosis of PC9GR cells induced by pitavastatin. However, the high expression of YAP inhibited the PC9 cells apoptosis induced by pitavastatin moderately (Fig. 5G–J). These results suggested that YAP was involved in the inhibitory effect of pitavastatin on lung cancer cells, especially in PC9GR cells. Furthermore, gefitinib was applied to PC9GR cells: the results showed that low expression of YAP further reduced the resistance to gefitinib in PC9GR cells inhibited by pitavastatin (Fig. 5K), while high expression of YAP significantly delayed the resistance to gefitinib in PC9GR cells inhibited by pitavastatin (Fig. 5L). This suggests that YAP signaling pathway plays a regulatory role in the reduction of PC9GR cell resistance to gefitinib by pitavastatin.

### YAP inteference inhibited the AKT-BAD-BCL-2 pathway in a pAKT-dependent manner

**Fig. 6:**
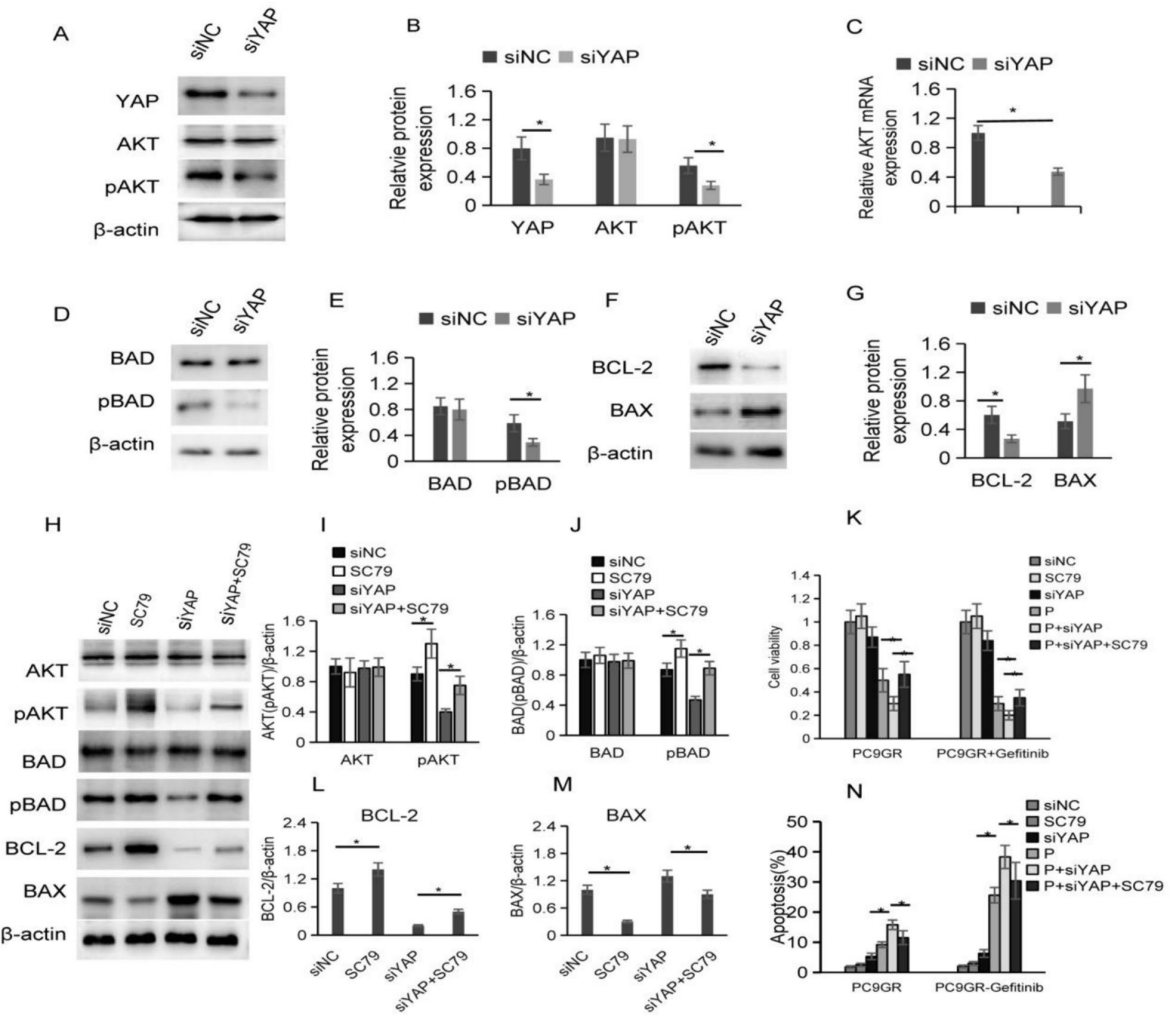
YAP inteference inhibited the AKT-BAD-BCL-2 pathway in a pAKT-dependent manner. **A**–**B** PC9GR cells were transfected with siYAP, and then YAP, AKT and pAKT protein levels were assessed by western blotting. **C** AKT mRNA levels decreased in YAP siRNA PC9GR cells. **D-E** BAD and pBAD protein levels. **F-G** BCL-2 and BAX protein expression. **H**–**L** The protein expression of AKT, pAKT, BAD, pBAD, BCL-2, BAX with the interaction of SC79 with siYAP. **M** Cell viability of siYAP, SC79 and pitavastatin combination on PC9GR cells after gefitinib treatments (*p ≤ 0.05). **N** Cell apoptosis of siYAP, SC79 and pitavastatin combination on PC9GR cells after gefitinib treatments (*p < 0.05)

To examine the impact of YAP on the treatment of TKI resistant PC9GR cells by pitavastatin, we examined the effect of YAP on AKT-BAD-BCL-2 pathway. It showed that YAP interference significantly inhibited AKT mRNA and pAKT/AKT protein expression, suggesting that YAP may regulate the expression of AKT/pAKT at the transcriptional level (Fig. 6A–C). We examined the levels of BAD, pBAD, BCL-2 and BAX and found that when YAP expression decreased, the levels of pBAD and BCL-2 were reduced, while the levels of BAX substantially elevated (Fig. 6D–G).

Next, the western blot showed that AKT phosphorylation inhibited by siYAP was elevated by SC79, and SC79 had no substantial impacts on the level of AKT and BAD. However, it showed that SC79 not only activated pAKT but also significantly raised the decrease of pBAD caused by the interference of YAP. At the same time, SC79 significantly increased BCL-2 and decreased BAX expression. Thus, the YAP-induced AKT-BAD-BCL-2 pathway is pAKT-dependent (Fig. 6H–L). Further experimental results showed that decresed YAP expression could increase the growth inhibition of PC9GR gefitinib resistant cells induced by pitavastatin (cell survival decreased in the pitavastatin + siYAP group compared with pitavastatin group); However, the effect of siYAP was significantly mitigated by SC79 (cell survival was significantly increased in the pitavastatin + siYAP + SC79 group compared to the pitavastatin + siYAP group)(Fig. 6M). Also, flow cytometry analysis showed that decreased YAP expression enhanced pitavastatin induced apoptosis in PC9GR gefitinib-resistant cells (increased apoptosis in the pitavastatin + siYAP group compared with the pitavastatin group), while the effect of siYAP was significantly reduced by SC79 (Fig. 6N). These results further confirmed that the YAP/AKT-BAD-BCL-2 pathway was involved in the inhibition of the survival of PC9GR gefitinib resistant cells by pitavastatin, and alleviated the resistance of PC9 resistant cells to gefitinib.

## Discussion

The current study reported that cholesterol-lowering drug pitavastatin enhances gefitinib therapy in lung cancer and alleviates acquired anti-EGFR resistance in gefitinib resistant cell lines. The effect of pitavastatin was mainly due to its ability to lower the activation of YAP/AKT/BAD-BCL-2 pathway signaling. Therefore, the study provides experimental evidence for the use of anti-metabolic agents to combat EGFR-TKI resistance in EGFR-mutated NSCLC patients.

Lung cancer is considered one of the most prevalent and deadly tumors globally. The use of tyrosine kinase inhibitors targeting EGF receptors has offered useful outcomes to patients. However, the progressive development of drug resistance has been a major challenge clinically, how to treat such patients needs to be done [30–32]. In this study, after the induction of drug resistance with a low dose of gefitinib, PC9 cells showed drug resistance. The targeted gene mutation detection found that there was no T790M mutation but a new mutation site NRAS mutation, for which there is no specific drug at present. During tumor progression, the levels or types of metabolites in tumor cells change, which promotes tumor growth by affecting gene expression, cell status, and tumor micro-environment. It has been demonstrated that reprogramming of metabolic cascades contributes significantly to drug resistance in cancer cells. [33, 34]. Pitavastatin is, therefore a known candidate antimetabolic agent for EGFR TKI resistant cell therapy.

Statins are cholesterol-lowering drugs, but studies have shown they have anti-cancer effects by anti-inflammation, angiogenesis inhibition and immune regulation. Statins potentially reduce the risk of mortality and improve the overall survival of lung cancer patients in observational studies [26]. In the present study, pitavastatin combined with gefitinib, as expected, the use of pitavastatin increases the susceptibility of PC9 cells to gefitinib and promotes cell apoptosis. Moreover, in the gefitinib-resistant PC9GR cells, the combination of pitavastatin reversed drug resistance, as demonstrated by CCK-8 and Ki67 assays. Notably, resistant cells exhibited greater sensitivity to pitavastatin in terms of cell growth and death as opposed to parental/sensitive cells.

Statins exert their lipid-lowering effects predominantly via the suppression of the rate-limiting enzyme 3-hydroxy-3-methylglutaryl Coenzyme reductase (HMGCR) in the mevalonate pathway. This pathway initiates with acetyl-CoA, which is produced when pyruvate, the final product of glycolysis, is decarboxylated [35]. Lipid metabolism can produce a large number of intermediates, such as mevalanoic acid, isoprene, etc., whose activity changes may affect their downstream signaling pathway disorders. Mevalonate is metabolized by a cascade of synthases and is metabolized to IPP, FPP and GGPP. The latter is required for post-translational isopropylation of small GTPases and contributes to several intracellular signaling pathways, which activate the YAP signaling pathway by suppressing their phosphorylation and accelerating their nuclear aggregation [36]. YAP pathway is a key oncogene in various cancer types, promoting the transcription of pro-growth genes and inhibiting pro-apoptosis genes. YAP is closely related to the resistance of traditional chemotherapy drugs. In this study, we conducted KEGG pathway analysis and confirmed that Hippo/ YAP signaling pathway has a very substantial expression change in drug-resistant cells. The YAP level of acquired resistant PC9GR cells was higher than that of parental PC9 cells, and the application of pitavastatin could down-regulate the level of YAP in PC9GR cells. The level of YAP was changed by gene transfection and RNA interference. The high expression of YAP significantly increased the resistance of PC9GR cells to gefitinib inhibited by pitavastatin, while the low expression of YAP significantly decreased the resistance of PC9GR cells to gefitinib. In line with us, some studies report the anti-tumor mechanism of pitavastatin, one of which is its suppressive effect on the YAP signaling pathway [37, 38].

Studies suggested that YAP may exert regulatory control over the PI3K-AKT pathway [39, 40]. The downstream target gene of the AKT signaling pathway regulates apoptosis and survival of cells [41]. In our study, it was noted that the upregulation of YAP remarkably increased the expression of pAKT/AKT and AKT mRNA, suggesting that YAP may promote the expression of AKT/pAKT at the transcriptional level. BAD, a BCL-2 homologous domain 3-related protein and a member of the BCL-2 protein family, may create a pro-apoptotic complex directly with the anti-apoptotic factor BCL-2. [42]. Ser-136 of the BAD protein is phosphorylated by AKT kinase [43]. The binding of 14-3-3 proteins to phosphorylated BAD results in the removal of BAD in the cytosol, thereby impeding its apoptotic progression across the mitochondrial membrane [44, 45]. The results of our study showed that when YAP was highly expressed, the level of pBAD-ser136 and anti-apoptotic protein BCL-2, were all substantially increased, and the pro-apoptotic protein BAX was decreased. The results also showed that YAP regulates the AKT-BAD-BCL-2 pathway in pAKT-dependent manner.

In summary, our study suggests that pitavastatin sensitizes the acquired resistance of EGFR-TKI in lung cancer via inhibiting YAP/AKT/BAD-BCL-2 pathway. Targeted metabolic reprogramming is a promising therapeutic approach for lung cancer combination therapy. Our study offers additional preclinical data and molecular basis regarding the combined use of antimetabolic and selective drugs in lung cancer.

### Supplementary Information

Supplementary material 1. Table S1. Primers of detected genes.

## Data Availability

All data and materials in this study are available from the corresponding author upon reasonable request.
