# Supplementary material for: Pitavastatin sensitizes the EGFR-TKI associated resistance in lung cancer by inhibiting YAP/AKT/BAD-BCL-2 pathway
Source: Cancer Cell Int. 2024 Jun 28;24:224. doi: 10.1186/s12935-024-03416-z (PMC11214206; doi:10.1186/s12935-024-03416-z)
Supplement: Supplementary file 1 — Supplementary material 1. Table S1. Primers of detected genes. [file 12935_2024_3416_MOESM1_ESM.docx]

### Supplementary material

Table1 Primers of detected genes

| Gene name | Sequence of forward and reverse primers (5ʹ to 3ʹ) | Fragment size |
| --- | --- | --- |
| MVK | AACCAGCACCATCTGAATGC  CCTGGCTTGAGGAGTGTGAT | 140 |
| SREBF2 | ACAAGTCTGGCGTTCTGAGG  ACCAGACTGCCTAGGTCGAT | 145 |
| LDLR | TGAACTGGTGTGAGAGGACC  CCTGACGGTGGATGTCTCCT | 194 |
| HMGCS1 | TGGGAATTGTTGCCCTTGAG  ACCACAGTCATGCAAAGAGAGT | 169 |
| ACACA | CGCCAGCTTAAGGACAACAC  GGGATGTTCCCTCTGTTTGGA | 80 |
| SCD1 | ACGATGCCCCTCTACTTGGA  GGGCTTGGGCCTTCCTTATC | 92 |
| FASN | CTCAGCCGCCATCTACAACA  GCCAGCGTCTTCCACACTAT | 129 |
| GPX4 | CAGTGAGGCAAGACCGAAGT  CCGAACTGGTTACACGGGAA | 104 |
| FADS2 | CCCCTGCTGATTGGTGAACT  CTCTCCAGGGCGATGATGTG | 173 |
| HMGCR | CATAGGAGGCTACAACGCCC  ACCACCCACCGTTCCTATCT | 187 |
| AKT | GCACTTTCGGCAAGGTGATC  CGTGGCCAAGGACGAGGTG | 101 |
| ACTIN | GAAGAGCTACGAGCTGCCTGA  CAGACAGCACTGTGTTGGCG | 191 |
